# “The devil is in the detail”: geographical inequalities of femicides in Ecuador

**DOI:** 10.1186/s12939-021-01454-x

**Published:** 2021-05-04

**Authors:** Osvaldo Fonseca-Rodríguez, Miguel San Sebastián

**Affiliations:** Department of Epidemiology and Global Health, Umeå University, Umeå, 901 87 Sweden

**Keywords:** Femicide, Spatial analysis, Gender, Violence, Ecuador

## Abstract

**Background:**

Femicide is a very important public health problem in Ecuador. Since regional and country-level femicide rates can obscure significant variations at the sub-national level, it is important to provide information at the lowest relevant level of disaggregation to be able to develop targeted preventive policies. The aim of this study was to assess the spatial distribution of the femicide rate and to examine its spatial clustering at the canton level in Ecuador in the period 2018–2019.

**Methods:**

Data on cases were collected by a national network of non-governmental organizations. Two age-disaggregated analyses were done, one for the 15 to 24 years-olds and the other for the female population of 15 and older. Age-specific population data were obtained from the National Institute of Statistics for the study period. Standardized mortality ratios for mapping the mortality were calculated using hierarchical Bayesian models and spatial scan statistics were applied to identify local clusters. Thematic maps of age-specific femicide rates were also constructed.

**Results:**

During the two-year period, 61 and 183 women were killed in the age ranges 15–24 and 15 years and older, respectively. The annual rate of femicides in Ecuador was 1.0 and 0.8 per 100,000 in the female population aged 15–24 and 15+, respectively, with substantial variations among cantons. The spatial analysis contributed to visualize high risk cantons, which were mainly located in a small area in the central part of the country (for those 15+) but especially in the Amazon region, for both of the studied age groups.

**Conclusions:**

This study has shown the usefulness of applying spatial analysis to the problem of femicides in Ecuador. The study has revealed important variations among cantons but also a spatial clustering, mainly in the Amazon region of the country. The results should help policymakers to focus on current prevention programmes for violence against women into these high-risk areas. Continuous monitoring of femicides at low-level geographical areas is highly recommended.

**Supplementary Information:**

The online version contains supplementary material available at 10.1186/s12939-021-01454-x.

## Introduction

Femicide, defined as the gender-based killing of women and girls, motivated by hatred, contempt, pleasure or a sense of ownership of women, has to be understood “in the context of the overall oppression of women in a patriarchal society” [1, 2]. Globally, a total of 87,000 women and girls were intentionally murdered in 2017, with more than half of them (58%) killed by intimate partners or family members [3]. In the past few decades activists have been struggling to raise visibility around femicides, leading to a series of policies and programmes using social, public health, and criminal justice responses at both national and international levels [3–6]. It seems, however, that more needs to be done.

More than half of the 25 countries with high and very high femicide rates (at least three femicides per 100,000 female population) in the world are in the Americas: 4 in the Caribbean, 4 in Central America, and 6 in South America [7]. According to the Gender Equality Observatory (GEO) of the Economic Commission for Latin America and the Caribbean, official information for 15 countries in the region shows that at least 3287 women were victims of femicide in 2018. The countries with the highest rate of femicide per 100,000 women were El Salvador (6.8), Honduras (5.1), Bolivia (2.3), and Guatemala (2.0). Ecuador reported a rate of 1.3 deaths per 100,000 women [8].

During the last decade, Ecuador has issued a series of progressive laws aiming to protect girls and women against all types of violence [9]. The last of these efforts was the 2018 Integral Organic Law to Prevent and Eradicate Violence against Women. In 2011, a special technical sub-commission for femicides validation was created under the National Institute of Statistics with the purpose of gathering, validating, and analyzing femicide information nationally. The official data from the last two years (2017–2018) included 103 and 60 deaths per year, respectively [10].

While these efforts are of great importance, concerns were raised by national non-governmental organizations (NGOs) working on gender-based violence that the official numbers might be biased due to the restricted definition of femicide that is applied. According to the official legal definition of femicide, only death of an adult woman committed by the intimate partner, are included as femicides. Deaths preceded by rape, sexual abuse by a non-intimate partner, when the killer commits suicide, or deaths of girls and adolescents (even those committed by an intimate partner) are therefore excluded in the legal definition of femicide used by the Ecuadorian government.

With this in mind, a national network of four non-governmental organizations (*Red Nacional de Casas de Acogida, Fundación Aldea, Comisión Ecuménica de Derechos Humanos, Taller Comunicación Mujer*, in Spanish) decided in 2017 to co-ordinate the collection of their own data on femicides using different sources and broadening the legal definition. The work is co-ordinated from the National Network of Shelters (NNS; *Red Nacional de Casas de Acogida* in Spanish) where the information is gathered and organized for advocacy purposes. Under this definition, the number of femicides reported by NNS in the last three years (2017–2019) was 155, 96 and 106 deaths per year, respectively.

This study builds on our previous research, where the situation with femicides at the provincial level in 2017, based on data from NNS, was described [11]. The femicide rate among women 15 years and above, 2.41cases/100,000, was one of the highest in the Latin American and the Caribbean region, with important variations among provinces. However, no available data at lower geographical level, such as cantons, was available at that time. Since then, the NNS has continued collecting information on femicides including the canton level.

Since regional and country-level femicide rates can obscure significant variations at the sub-national level, it is important to provide information at the lowest relevant level of disaggregation to be able to develop targeted preventive policies. In this sense, spatial analysis can be a particularly valuable tool for identifying health disparities across a geographic region, allowing policymakers to prioritize communities for intervention efforts [12, 13]. Therefore, the aim of this study was to assess the spatial distribution of the femicide rate and to examine its spatial clustering at the canton level in Ecuador in the period 2018–2019.

## Methods

### Study area and data sources

The study area was mainland Ecuador, which currently includes 23 provinces and 218 cantons. The Galapagos Islands (with three cantons) were excluded from the analysis due to the lack of geographical continuity with the rest of the country. In the risk-mapping process, the spatial continuity of risk is a common assumption [14], and the inclusion in the analyses of those distant territories can bias the results.

As previously described [11], data on femicides were collected by the national network of NGOs and sent it to the NNS using different sources: two national non-governmental organizations (the network of shelters for women victims of violence and the network of external care centers for women victims of violence) and the continuous review of local and national media. NNS also validates the cases with the local prosecutor’s office and with the judiciary council. An external lawyer advises NNS in cases where there is a doubt about quality control. The definition of femicide used by the NNS refers to any murder of a woman for gender-based motivations (such as hatred, contempt, pleasure or a sense of ownership over women), including all ages and deaths committed by intimate and non-intimate persons. Name, age, date, place of event (province, canton), nature of the relationship between the victim and perpetrator and weapon used were recorded in the NNS database, which was anonymized for analytical purposes. This database is not publicly available and any request should be addressed to NNS.

Age-specific population data were obtained from projections carried out by the National Institute of Statistics and Census (*INEC* in Spanish) for the years 2018–19, based on the national census of 2010. Three databases were combined: the sex and age distribution of the cantons in 2010 and the projection of the total population by canton in 2018 and 2019 applied to the previous sex and age datasets. These datasets are publicly available at the website of the INEC.

### Statistical analysis

Two age disaggregated analyses were done, one for the 15 to 24 years-olds and the other for the female population of 15 and older. A descriptive analysis of the study population was first performed. To achieve the first objective, the standardized mortality ratio (SMR) for mapping the mortality (femicides) was calculated following the indirect standardization method. The number of observed femicides by age group in each canton (*Oi*) was standardized across the study area. For this purpose, the observed femicides in Ecuador over the two years was divided by the annual national population (2018–2019). This value was multiplied by the population of each canton to estimate the expected (*Ei*) local cases. Thus, the SMR was calculated as follows:

$$ SMR=\frac{Oi}{Ei} $$

The 95% confidence interval (95% CI) of the SMR by canton was estimated using the function “epi.conf” of the package “epiR” version 1.0–14 in R software, version 3.6.0 [15]. The results of the SMR and 95% CI are shown in Fig. S1 and Table S1.

However, the SMR is highly dependent on population size; thus, in areas with a small population, such as in some of the cantons, it could be challenging to use this method for estimating the risk since it may produce unstable risk estimates with high variance [16, 17]. Thus, in order to improve the stability of the estimation by cantons with small populations, a hierarchical Bayesian model to smooth the SMR (sSMR) was applied. Hence, the spatial autoregressive model proposed by Besag, York and Mollié [18] was performed, and estimations of sSMR were developed using the Integrated Nested Laplace Approximation implemented in R-INLA package [19].

The second objective aimed to identify spatial clusters of femicides in Ecuador. To achieve this, a spatial analysis using the scan statistics method implemented in SaTScan™ 9.6 was performed [20]. This is a local cluster test that identifies the clustered area and its statistical significance. This is done by gradually scanning using a spatial window of different sizes across the study area, adjusting for the dissimilar geographical distribution of the population, and conditioned on the total observed cases [21].

When the spatial clusters are exceptionally large, they cannot be useful from the practical point of view, and it is essential to identify specific areas of higher importance inside the clusters. These core areas are of more relevance from research and policy perspectives [22]. Thus, the retrospective isotonic spatial scan statistic implemented in SaTScan™ 9.6 [20, 23] was used to detect the spatial clusters and to reveal different levels of risk inside the clusters. The isotonic scan statistic produces several steps corresponding to the multiple circular spatial windows [23]. Those circles were centralized in the initially identified canton as a centre of the cluster, and the steps in the risk function allowed us to verify the differences of the case intensity within the high-risk areas [24]. Thus, the highest rate is within the central circle and continues decreasing until the outer circle [23, 24].

This approach has demonstrated to be a proper method to analyze the spatial clustering of territorial subunits with rare events [24]. The femicide cases by canton occurred between 2018 and 2019, and the mean estimated female population by canton according to the different age ranges was considered in the analysis. Thus, the Poisson probability model and 9999 replications were used to detect high-rates clusters of femicides by age range. The default option for defining the maximum cluster size was 50% of the population at risk, as suggested by Kulldorff [20]. However, since it could produce considerable large clusters that might not be useful for implementing some interventions [22], the maximum size of the spatial window was determined at 20% of the population at risk. Thus, the geographic dimension of the cluster was reduced while considering that the most populated canton in Ecuador had around 17% of the population at risk in both study groups. Cartographical displays of the results were performed using R software, version 3.6.0 [15].

## Results

The characteristics of the study populations by cantons (*N* = 218) are shown in Table 1. During the two years period, 61 and 183 women were killed in the age ranges 15–24 and 15 years and older, respectively. The annual rate of femicides in Ecuador was 1.0 per 100,000 and 0.8 per 100,000 in the female population aged 15–24 and 15+, respectively, with substantial variations among cantons (Fig. S1 and Table S1).

**Table 1:** Characteristics of the study population of 218 cantons from mainland Ecuador 2018–2019.

Age Range		Minimum	Percentile 25th	Median	Percentile 75th	Maximum
15–24 years	Cases	0	0	0	0	8
	Population*	312	2240	4946	10,678	504,568
> 14 years	Cases	0	0	0	1	19
	Population*	1427	8031	17,221	37,452	1,951,216

*Sum of the annual populations (2018–2019)

**Fig. 1:**
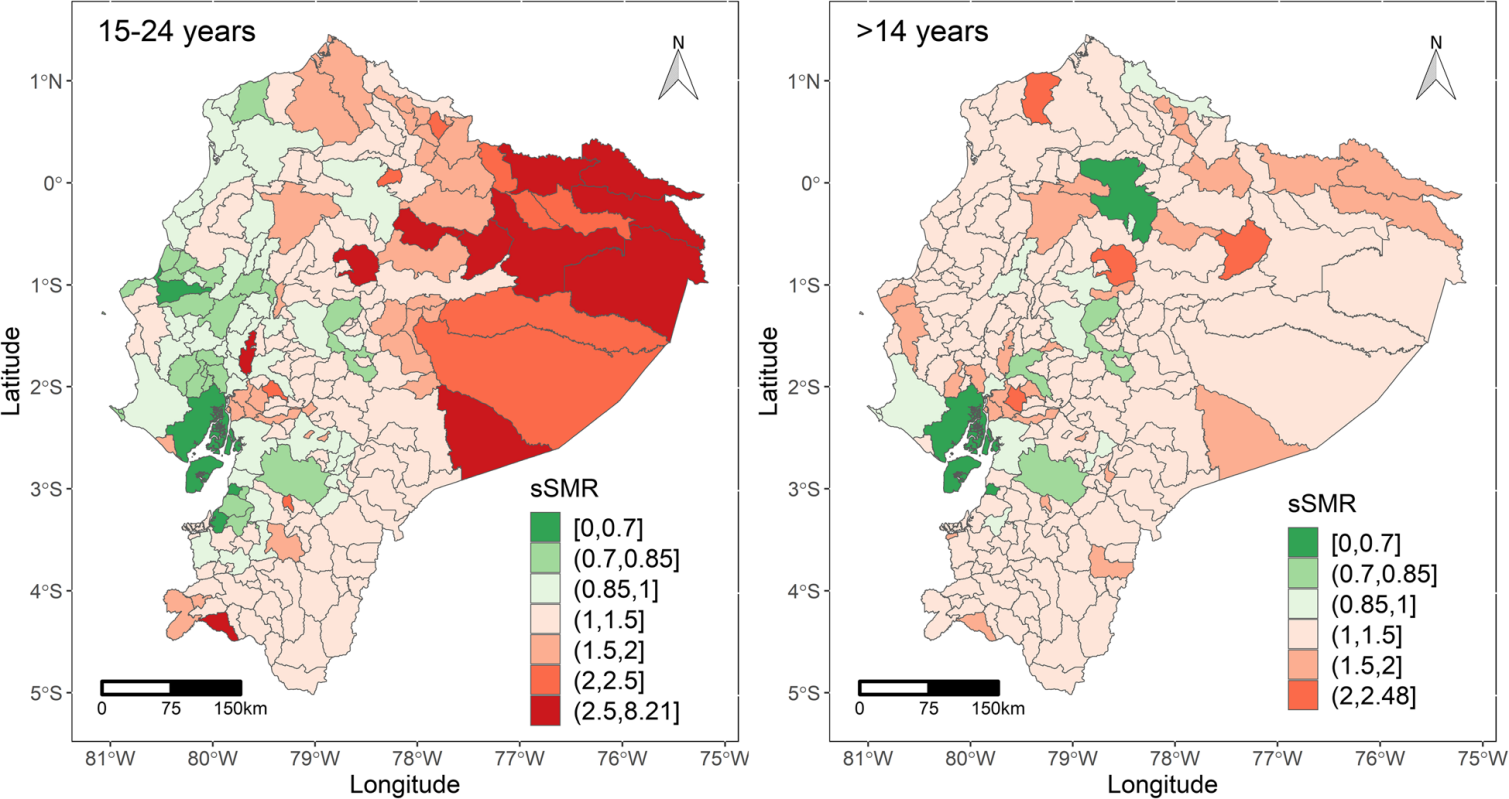
Smoothed Standardised Mortality Ratio (sSMR) of femicides among women 15–24 years old (left) and > 14 years old (right) by cantons. Their respective probability of sSMR exceeded one is shown in the maps at the bottom, left and right

When the mortality ratios were smoothed, the sSMR ranged from 0.45 to 8.20 and from 0.58 to 2.48 among women aged 15–24 and > 14, respectively. Femicides in Ecuador showed a marked pattern of geographical distribution in mainly in women aged 15–24 but it is not so evident for women population > 14 years old, as shown in Fig. 1. Ratios greater than one indicate that the mortality was higher than expected. Overall, the femicide ratios were higher in the northern and eastern parts of the country, particularly in the 15–24 age group, while being more scattered in the 15 years and older age group. Also, in both groups, lower mortality ratios were more common in western and coastal areas, and this pattern was more evident for the younger population.

The spatial scan statistics analysis detected one high-risk area for femicides in women aged 15 to 24 years in the northeastern part of the country. The annual rate of femicides in this cluster was 3.1 per 100,000, the highest among the clustered areas, with an overall risk of femicides of 4.37 times higher compared to the rest of the country. This cluster showed a coincidence of 75.61% (31 out of 41) of cantons with the spatial cluster reported for the wider age range group of women (> 14 years).

**Fig. 2:**
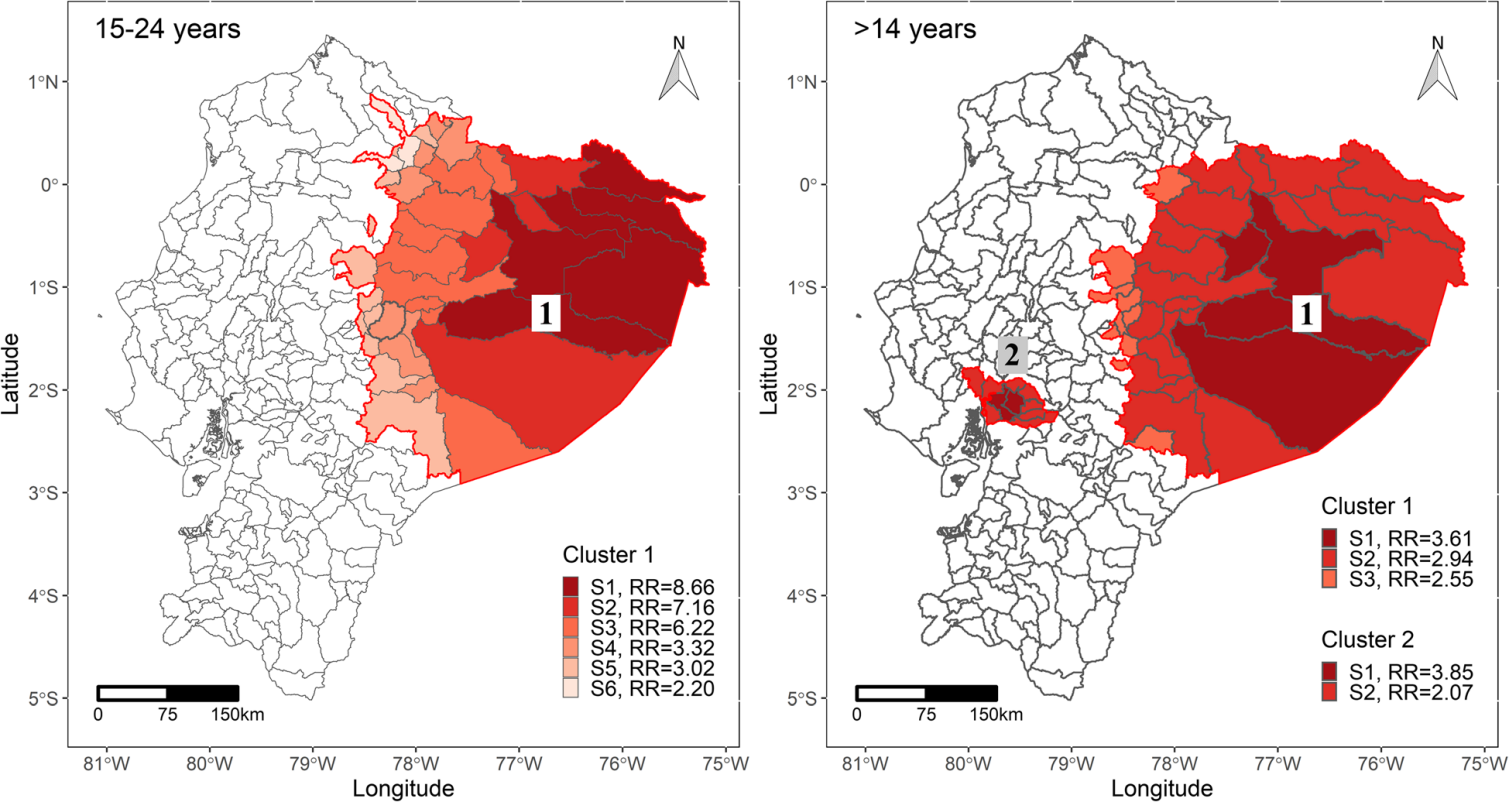
High-risk spatial clusters of femicides by canton (highlighted in red tones by step) in Ecuador (2018–2019). Femicides in the population between 15 and 24 (left) and > 14 years old (right). The numbers in the squares identify the clusters in the Fig. S1 to S6 shows the number of steps in the risk function identified through isotonic spatial scan statistics. RR is the relative risk in each step. Complementary information is available in Table 2

Two high-risk clusters of femicides in women aged > 14 years were identified in the northeastern and central parts of the country. Cluster 1 included 36 cantons where the annual rate of deaths was 2.0 femicides per 100,000 women, and the risk of femicide occurrence 2.85 higher than in the rest of the country (Fig. 2, Table 2). Cluster two grouped 10 locations in which 25 femicides occurred with an annual rate of 1.9 per 100,000 and a risk of femicide 2.54 times higher than outside the cluster (Fig. 2, Table 2).

**Table 2:** Characteristics of the spatial clusters of femicides in Ecuador (2018–2019). Overall result.

Age range	Cluster	Steps	Radius (km)	Number cantons	LLR	*p*-value	Population	Observed (O)	Expected (E)	O/E	RR (95%CI)
15–24 years	1	6	301.36	41	14.65	> 0.001	336,928	21	6.55	3.21	4.37 (2.57–7.40)
> 14 years	1	3	211.28	36	12.06	0.002	822,984	33	13.10	2.52	2.85 (1.96–4.16)
	2	2	39.98	10	8.59	0.038	673,327	25	10.72	2.33	2.54 (1.67–3.88)

LLR – Log-Likelihood Ratio; O/E – observed cases divided by the expected cases; RR – Relative risk, CI – Confidence interval

## Discussion

This study has shown the rates of femicides in the 15–24 (1/100,000) and 15 years and old (0.8/100,000) groups in Ecuador. The figures reveal a serious public health concern, particularly for young women. The observed rates are lower than those from 2017 when, using the same source, the femicide rate (× 100,000) in the youth (15–24 years) was 3.78 and 2.41 in the 15+ age group [14]. While the reduction might reflect a positive trend, the increase in the number of cases in 2019 compared to 2018 calls for closer monitoring.

While the national average rate of femicides puts Ecuador in a moderate risk position compared to some central American countries or Brazil and Colombia in the Latin American region [8, 25, 26], the picture changes when disaggregated information is assessed. Femicide rates of two to eight per 100,000 women were observed in several of the cantons, being among the highest in the world. The spatial analysis contributed to visualize those highly risk cantons which were mainly located in a small area in the central part of the country (for those 15+) but specially in the Amazon region, for the two studied age groups.

The last National Survey of Family Relations and Gender Violence against Women in Ecuador conducted in 2019 reported a higher prevalence of physical and psychological violence in the Amazon compared to the other regions in the country [27]. Contexts of increase violence, particularly in abusive relationships, have been linked to femicides in different studies [28, 29]. The Amazon region has been exposed to oil exploitation since the 1970s and, more recently, to different kinds of mineral extraction, creating a series of socio-environmental conflicts over the years [30, 31]. It is well known that both types of extractive industries can contribute to generate conflicts with the communities, structural changes in employment, demography and families, to increase the use of alcohol, drugs and prostitution as well as violence against women [32, 33]. The role of these extractive industries as potential drivers of gender-based violence and femicides would require a deeper investigation.

### Methodological considerations

Some methodological considerations should also be mentioned. Although the duplication of cases could be a source of overestimation, all cases were collected with their names by the NNS, avoiding this potential problem. Due to the lack of data, the calculation of the cantons´ population was based on crude projections based on the sex and age distribution of the population in the census of 2010. The size of the error leading to an over- or underestimation of the rates could not be calculated.

As this and previous studies illustrate [14], narrow legal definitions of femicides used by governmental institutions compared to the broader definitions used by NGOs can create enormous differences on the reported rates of femicide, potentially hiding the true number of women’s deaths that have occurred. In addition, and despite the broad definition and rigorous search for cases by the NNS, our findings most likely underestimate the female homicide rate because of the difficulty in finding cases. Some differences in reporting by cantons could also be present due to a more active involvement in some of the regions by individual NGOs, leading to a certain variation in the calculation of the rates of some cantons. The role of this error was, however, not possible to assess.

No socioeconomic information from the cantons or the women was available, hindering the possibility of assessing potential risk factors for femicides. Further investigations should include relevant sociodemographic and socioeconomic variables that could be associated with the cluster areas.

Finally, scan statistics also involve certain limitations. The results are sensitive to the parameter settings in running SaTScan, such as modifying the size of the study area or the maximum size of the spatial windows [22, 34, 35]. However, our area of investigation was the entire country, and after modifying the size of the spatial windows (50, 40, 30, and 20%), our results were consistent. Another limitation of the scan statistic relates to the detection of irregular shaped clusters due to its circular scan window [36, 37], which could decrease statistical power [38].

## Conclusion

Femicides continue to be a highly important public health problem in Ecuador. This study has shown the usefulness of applying a spatial analysis to this problem. The study has revealed important variations among cantons but also a clustering mainly in the Amazon region of the country. The results should help policymakers to focus the violence against women prevention programmes in these high-risk areas. A continuous monitoring of femicides at low-level geographical areas is highly recommended.

## Supplementary Information

**Additional file 1 Fig. S1.** Cartographical display of Standardized Mortality Ratio (SMR) of femicides by cantons. **Table S1.** Standardized Mortality Ratio (SMR) and 95% confidence intervals (CI) of femicides by cantons.

## Data Availability

The data that support the findings of this study are available from the National Network of Shelters but restrictions apply to the availability of these data, which were used under license for the current study, and so are not publicly available. Data are however available from the authors upon reasonable request and with permission of National Network of Shelters in Ecuador.

## Abbreviations

GEO: Gender Equality Observatory
NGO: Non-Governmental Organization
NNS: National Network of Shelters
INEC: National Institute of Statistics and Census
SMR: Standardized Mortality Ratio
Oi: Observed
Ei: Expected
CI: confidence interval
sSMR: smooth Standardized Mortality Ratio
LLR: Log-Likelihood Ratio
RR: Relative risk
