# Supplementary material for: “The devil is in the detail”: geographical inequalities of femicides in Ecuador
Source: Int J Equity Health. 2021 May 4;20:115. doi: 10.1186/s12939-021-01454-x (PMC8097816; doi:10.1186/s12939-021-01454-x)
Supplement: Supplementary file 1 — Additional file 1 Fig. S1. Cartographical display of Standardized Mortality Ratio (SMR) of femicides by cantons. Table S1. Standardized Mortality Ratio (SMR) and 95% confidence intervals (CI) of femicides by cantons. [file 12939_2021_1454_MOESM1_ESM.pdf]

Supplementary material

"The devil is in the detail": geographical inequalities of femicides in Ecuador

Authors:
Osvaldo Fonseca-Rodríguez[1, +]
Miguel San Sebastián[1]

[1] Department of Epidemiology and Global Health, Umeå University, 901 87 Umeå, Sweden

[+] Corresponding author
osvaldo.fonseca@umu.se

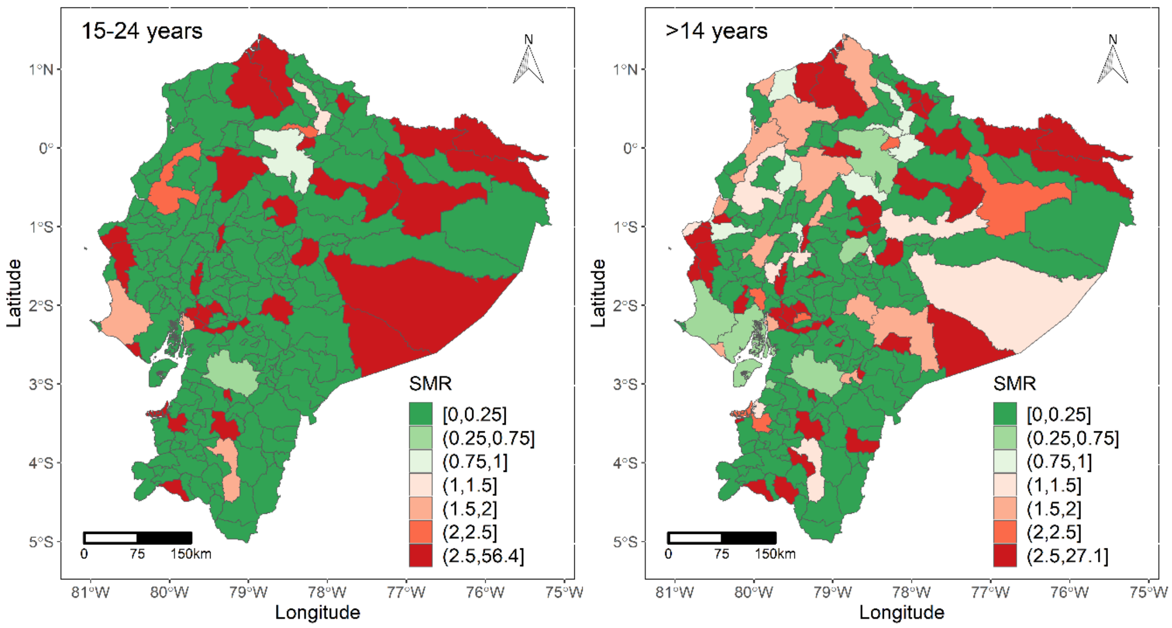

Fig. S1. Cartographical display of Standardized Mortality Ratio (SMR) of femicides by cantons

Table. S1. Standardized Mortality Ratio (SMR) and 95% confidence intervals (CI) of femicides by cantons

CANTON	15 - 24 years			>14 years		
	SMR	95%CI		SMR	95%CI	
		lower	upper		lower	upper
**CUENCA**	0.4	0.01	2.23	0.56	0.15	1.44
**GIRON**	0	0	71.95	0	0	24.86
**GUALACEO**	0	0	17.73	1.89	0.05	10.55
**NABON**	0	0	61.87	0	0	20.56
**PAUTE**	0	0	32.02	0	0	11.59
**PUCARA**	0	0	108.69	0	0	36.14
**SAN FERNANDO**	56.44	1.43	314.46	19.98	0.51	111.32
**SANTA ISABEL**	0	0	46.47	0	0	16.79
**SIGSIG**	0	0	31.11	0	0	11.25
**OÑA**	0	0	271.3	0	0	80.56
**CHORDELEG**	0	0	58.59	0	0	22.55
**EL PAN**	0	0	398.67	27.08	0.69	150.86
**SEVILLA DE ORO**	0	0	159.68	0	0	50.95
**GUACHAPALA**	0	0	261.23	0	0	83.41
**CAMILO PONCE ENRIQUEZ**	0	0	30.04	0	0	11.73
**GUARANDA**	0	0	9.66	0	0	3.38
**CHILLANES**	0	0	69.09	0	0	21.82
**SAN JOSE DE CHIMBO**	0	0	64.94	0	0	19.56
**ECHEANDIA**	0	0	83.71	0	0	26.15
**SAN MIGUEL**	0	0	36.49	0	0	11.62
**CALUMA**	0	0	68.46	5.91	0.15	32.93
**LAS NAVES**	0	0	148.21	0	0	51.4
**AZOGUES**	0	0	10.58	0	0	3.81
**BIBLIAN**	0	0	37.56	0	0	13.28
**CAÑAR**	0	0	13.48	0	0	5.11
**LA TRONCAL**	0	0	13.58	0	0	5.04
**EL TAMBO**	0	0	70.83	7.84	0.2	43.66
**DELEG**	0	0	155.88	0	0	43.91
**SUSCAL**	0	0	132.09	0	0	53.64
**TULCAN**	0	0	10.63	0	0	3.39
**BOLIVAR (CARCHI)**	0	0	77.48	6.16	0.16	34.3
**ESPEJO**	0	0	81.66	6.62	0.17	36.86
**MIRA**	0	0	93.65	0	0	28.94
**MONTUFAR**	8.53	0.22	47.51	2.7	0.07	15.02
**SAN PEDRO DE HUACA**	0	0	120.93	0	0	39.7
**LATACUNGA**	5.27	1.44	13.5	3.17	1.27	6.52
**LA MANA**	0	0	18.63	1.83	0.05	10.2
**PANGUA**	0	0	47.8	0	0	15.94
**PUJILI**	0	0	12.72	0	0	4.64
**SALCEDO**	0	0	14.69	4.07	0.84	11.89

**SAQUISILI**	0	0	30.96	0	0	11.69
**SIGCHOS**	0	0	49.9	0	0	17.33
**RIOBAMBA**	0	0	3.58	0	0	1.23
**ALAUSI**	0	0	22.98	0	0	8.35
**COLTA**	0	0	24.04	0	0	7.3
**CHAMBO**	0	0	71.41	0	0	25.13
**CHUNCHI**	0	0	77.83	0	0	28.18
**GUAMOTE**	4.87	0.12	27.11	1.89	0.05	10.54
**GUANO**	0	0	20.14	0	0	7.07
**PALLATANGA**	0	0	91.52	0	0	30.51
**PENIPE**	0	0	176.64	0	0	45.83
**CUMANDA**	16.08	0.41	89.59	5.68	0.14	31.66
**MACHALA**	0	0	3.69	1.3	0.35	3.33
**ARENILLAS**	0	0	32.34	0	0	11.05
**ATAHUALPA**	0	0	201.12	0	0	52.64
**BALSAS**	0	0	123.31	0	0	41.5
**CHILLA**	0	0	609.31	0	0	157.26
**EL GUABO**	0	0	17.52	0	0	6.13
**HUAQUILLAS**	0	0	17.35	4.99	1.03	14.58
**MARCABELI**	0	0	182.93	0	0	59.47
**PASAJE**	0	0	12.36	0	0	4.02
**PIÑAS**	0	0	39.13	0	0	11.37
**PORTOVELO**	0	0	81.9	0	0	25.2
**SANTA ROSA**	3.59	0.09	20.03	2.35	0.28	8.5
**ZARUMA**	0	0	47.3	0	0	13.43
**LAS LAJAS**	0	0	242.71	0	0	71.09
**ESMERALDAS**	0	0	4.58	0.88	0.11	3.19
**ELOY ALFARO**	7.03	0.18	39.14	2.61	0.07	14.55
**MUISNE**	0	0	38.31	0	0	13.86
**QUININDE**	0	0	7.57	1.53	0.18	5.51
**SAN LORENZO**	4.88	0.12	27.2	1.86	0.05	10.38
**ATACAMES**	0	0	19.56	1.97	0.05	10.96
**RIOVERDE**	0	0	36.69	11.14	2.3	32.56
**LA CONCORDIA**	0	0	20.27	0	0	7.42
**GUAYAQUIL**	0.11	0	0.6	0.47	0.26	0.79
**ALFREDO BAQUERIZO MORENO**	0	0	36.85	0	0	11.93
**BALAO**	0	0	42.98	0	0	15.27
**BALZAR**	0	0	18.72	1.75	0.04	9.77
**COLIMES**	0	0	46.1	0	0	15.07
**DAULE**	0	0	7.17	2.27	0.62	5.81
**DURAN**	1.89	0.23	6.83	1.83	0.67	3.98
**EL EMPALME**	0	0	13.25	0	0	4.48
**EL TRIUNFO**	5	0.13	27.86	3.56	0.43	12.87
**MILAGRO**	3.04	0.37	10.97	3.84	1.66	7.56

**NARANJAL**	0	0	11.73	0	0	4.23
**NARANJITO**	0	0	24.71	2.27	0.06	12.62
**PALESTINA**	0	0	63.14	0	0	20.5
**PEDRO CARBO**	0	0	22.53	0	0	7.46
**SAMBORONDON**	0	0	11.44	0	0	3.42
**SANTA LUCIA**	0	0	27.12	0	0	8.25
**URBINA JADO**	0	0	18.78	0	0	5.77
**YAGUACHI**	4.03	0.1	22.44	2.68	0.32	9.66
**PLAYAS**	5.21	0.13	29	1.72	0.04	9.61
**SIMON BOLIVAR**	10.05	0.25	55.97	6.44	0.78	23.28
**CORONEL MARCELINO MARIDUEÑA**	0	0	90.37	0	0	27.42
**LOMAS DE SARGENTILLO**	0	0	50.08	0	0	15.76
**NOBOL**	0	0	45.21	0	0	14.23
**GENERAL ANTONIO ELIZALDE**	0	0	86.52	0	0	28.72
**ISIDRO AYORA**	0	0	79.7	7.46	0.19	41.54
**IBARRA**	1.29	0.03	7.19	0.83	0.1	3
**ANTONIO ANTE**	0	0	19.24	0	0	6.4
**COTACACHI**	0	0	25.18	0	0	8.62
**OTAVALO**	2.17	0.06	12.11	0.78	0.02	4.33
**PIMAMPIRO**	0	0	82	0	0	26.12
**SAN MIGUEL DE URCUQUI**	0	0	62.14	0	0	20.62
**LOJA**	1.85	0.22	6.69	1.01	0.21	2.95
**CALVAS**	0	0	34.68	3.23	0.08	18
**CATAMAYO**	0	0	29.4	2.73	0.07	15.24
**CELICA**	0	0	70.86	0	0	23.79
**CHAGUARPAMBA**	0	0	210.21	0	0	54.2
**ESPINDOLA**	0	0	82.69	0	0	26.77
**GONZANAMA**	0	0	115.55	0	0	31.25
**MACARA**	14.12	0.36	78.7	9.61	1.16	34.7
**PALTAS**	0	0	53.19	0	0	15.28
**PUYANGO**	0	0	72.78	0	0	22.47
**SARAGURO**	8.73	0.22	48.64	2.97	0.08	16.52
**SOZORANGA**	0	0	166.23	0	0	51.11
**ZAPOTILLO**	0	0	85.09	0	0	26.79
**PINDAL**	0	0	109.61	0	0	37.09
**QUILANGA**	0	0	319.33	0	0	83.08
**OLMEDO (LOJA)**	0	0	297.44	0	0	81.28
**BABAHOYO**	0	0	6.25	0	0	2.05
**BABA**	14.04	1.7	50.71	4.77	0.58	17.24
**MONTALVO**	0	0	39.84	0	0	12.57
**PUEBLOVIEJO**	0	0	23.54	0	0	8.59
**QUEVEDO**	0	0	4.96	0.92	0.11	3.33
**URDANETA**	0	0	35.54	0	0	11.38

**VENTANAS**	0	0	15.11	1.37	0.03	7.61
**VINCES**	0	0	14.41	1.23	0.03	6.86
**PALENQUE**	0	0	51.14	0	0	16.65
**BUENA FE**	0	0	13.13	0	0	4.76
**VALENCIA**	0	0	20.95	0	0	7.41
**MOCACHE**	0	0	26.51	0	0	8.89
**QUINSALOMA**	15.02	0.38	83.69	5.25	0.13	29.27
**PORTOVIEJO**	0	0	3.31	0.87	0.18	2.53
**BOLIVAR (MANABÍ)**	0	0	23.13	0	0	7.98
**CHONE**	2.22	0.06	12.36	1.48	0.18	5.35
**EL CARMEN**	0	0	9.72	0.94	0.02	5.26
**FLAVIO ALFARO**	0	0	47.2	0	0	15.92
**JIPIJAPA**	4.22	0.11	23.52	2.56	0.31	9.25
**JUNIN**	0	0	58.11	0	0	18.88
**MANTA**	0	0	3.96	1.06	0.22	3.11
**MONTECRISTI**	2.87	0.07	15.97	2.86	0.59	8.37
**PAJAN**	0	0	33.15	0	0	10.16
**PICHINCHA**	0	0	38.46	0	0	13.42
**ROCAFUERTE**	0	0	30.35	2.55	0.06	14.21
**SANTA ANA**	0	0	23.02	0	0	7.57
**SUCRE (SAN ISIDRO)**	0	0	17.92	1.54	0.04	8.59
**SUCRE**	0	0	17.92	1.54	0.04	8.59
**TOSAGUA**	0	0	25.93	0	0	8.57
**24 DE MAYO**	0	0	41.2	0	0	12.87
**PEDERNALES**	0	0	17.45	1.82	0.05	10.12
**OLMEDO (MANABÍ)**	0	0	120.72	0	0	37.24
**PUERTO LOPEZ**	0	0	46.38	4.27	0.11	23.78
**JAMA**	0	0	37.14	0	0	14.85
**JARAMIJO**	0	0	36.85	3.91	0.1	21.79
**SAN VICENTE**	0	0	45.3	0	0	14.97
**MORONA**	0	0	17.08	1.92	0.05	10.67
**GUALAQUIZA**	0	0	50.06	0	0	20.95
**LIMON INDANZA**	0	0	100.9	0	0	39.05
**PALORA**	0	0	136.13	0	0	53.63
**SANTIAGO**	0	0	95.58	0	0	38.2
**SUCUA**	0	0	41.55	4.6	0.12	25.64
**HUAMBOYA**	0	0	91.12	0	0	40.98
**SAN JUAN BOSCO**	0	0	228.66	0	0	87.32
**TAISHA**	22.38	2.71	80.86	10.4	1.26	37.57
**LOGROÑO**	0	0	137.15	0	0	61.12
**PABLO VI**	0	0	400.6	0	0	162.38
**TIWINTZA**	0	0	115.02	0	0	47.24
**TENA**	0	0	13.02	1.42	0.04	7.92
**ARCHIDONA**	0	0	32.36	0	0	12.94

**EL CHACO**	0	0	114.17	0	0	39.12
**QUIJOS**	43.02	1.09	239.68	14.06	0.36	78.36
**CARLOS JULIO AROSEMENA**	0	0	262.16	0	0	97.1
**PASTAZA**	3.27	0.08	18.22	1.27	0.03	7.07
**MERA**	0	0	55.27	0	0	22.71
**SANTA CLARA**	0	0	250.7	0	0	100.29
**ARAJUNO**	0	0	150.11	0	0	62.84
**QUITO**	0.82	0.35	1.61	0.61	0.37	0.96
**CAYAMBE**	0	0	9.42	0.92	0.02	5.15
**MEJIA**	0	0	9.57	0.88	0.02	4.93
**PEDRO MONCAYO**	6.42	0.16	35.77	2.35	0.06	13.11
**RUMIÑAHUI**	0	0	9.22	0	0	2.86
**SAN MIGUEL DE LOS BANCOS**	0	0	36.89	7.56	0.92	27.3
**PEDRO VICENTE MALDONADO**	0	0	24.73	0	0	9.07
**PUERTO QUITO**	0	0	45.44	0	0	16.53
**AMBATO**	0	0	2.66	0.46	0.06	1.68
**BAÑOS**	11.45	0.29	63.78	3.66	0.09	20.38
**CEVALLOS**	0	0	113.01	9.04	0.23	50.39
**MOCHA**	0	0	151.64	0	0	43.98
**PATATE**	0	0	66.26	0	0	22.17
**QUERO**	0	0	48.33	0	0	16.39
**SAN PEDRO DE PELILEO**	0	0	15.13	1.36	0.03	7.55
**SANTIAGO DE PILLARO**	0	0	23.49	0	0	7.34
**TISALEO**	0	0	73.49	0	0	23.06
**ZAMORA**	0	0	30.68	0	0	12
**CHINCHIPE**	0	0	103.94	0	0	39.8
**NANGARITZA**	0	0	152.81	0	0	58.82
**YACUAMBI**	0	0	144.05	0	0	61.99
**YANTZAZA**	0	0	38.82	8.53	1.03	30.82
**EL PANGUI**	0	0	96.46	0	0	39.41
**CENTINELA DEL CONDOR**	0	0	128.94	0	0	50.81
**PALANDA**	0	0	112.94	0	0	43.43
**PAQUISHA**	0	0	186.32	0	0	79.63
**LAGO AGRIO**	4.75	0.58	17.15	3.57	0.97	9.15
**GONZALO PIZARRO**	0	0	108.91	11.13	0.28	62.01
**PUTUMAYO**	21.22	0.54	118.22	7.89	0.2	43.94
**SHUSHUFINDI**	0	0	18.8	0	0	7.24
**SUCUMBIOS**	0	0	275.63	0	0	101.13
**CASCALES**	0	0	71.72	0	0	27.62
**CUYABENO**	47.52	1.2	264.78	16.59	0.42	92.43
**ORELLANA**	6.13	0.74	22.15	2.4	0.29	8.68
**AGUARICO**	0	0	277.1	0	0	116.07
**LA JOYA DE LOS SACHAS**	0	0	26.19	0	0	10.04

**LORETO**	24.05	2.91	86.86	14.88	3.07	43.49
**SANTO DOMINGO DE LOS TSACHILAS**	3.02	0.98	7.05	1.74	0.75	3.43
**SANTA ELENA**	1.64	0.04	9.16	0.55	0.01	3.04
**LIBERTAD**	0	0	9.22	0	0	3.15
**SALINAS**	0	0	11.44	0	0	4.1
**LAS GOLONDRINAS**	0	0	128.02	0	0	88.94
**MANGA DEL CURA**	0	0	46.87	0	0	32.23
**EL PIEDRERO**	0	0	171.04	0	0	129.64
